# Dr. Paris, on the Effects of Muriatic Acid

**Published:** 1810-01

**Authors:** John Ayrton Paris

**Affiliations:** Westminster


### *To the Editors of the Medical and Physical Journal.*

GENTLEMEN,

THE extensive circulation which your valuable Journal enjoys in this kingdom, and the universal avidity with which it is consulted, renders it the most proper channel for imparting any information to the medical practitioner, remotely resident from the metropolis. It is with this view I beg leave to communicate to you, a history of the beneficial effects of muriatic acid in fevers. I have profusely exhibited it in the hospital to which I am physician, and the result has been highly important and decisive.

The *antiseptic* and *febrifugous* effects of acids have been well known and acknowledged from the earliest period of medical history; the extent, however, of their powers, when copiously employed, seems to be but recently understood; and the superiority of *muriatic acid* over the rest of this class of medicines is a truth of still later discovery.

In fevers of the *typhoid* type, and in the varieties of *scarlatina*, this medicine appears to be most prominently beneficial; for whilst its antiseptic virtues correct and destroy that tendency to putrescency, so characteristic of these diseases, its tonic powers afford considerable support and vigor to the system; and in the *scarlatina anginosa*, the detergent quality of the acid renders it a remedy still more valuable.

The

The phenomena to which the copious exhibition of muriatic acid gives birth, are an immediate change in the strength and number of the arterial pulsations; the weak, frequent, and often intermittent beat, becomes stronger, slower, and more regular; the animal temperature sinks; the thirst is assuaged; the excretions become more regular and healthy, and the increased vigor of the system is indicated by the feelings expressed by the patient, and a character and air of liveliness, which the face and actions assume. In order, however, to insure results so beneficial and immediate, the muriatic acid must be exhibited in very large doses. I generally direct two drachms to be diluted with a quart of barley-water, and sweetened with honey, as the proper quantity for twenty-four hours: previous to its exhibition, the bowels should be always evacuated by a saline purge; for I think I have more than once observed, that where it was neglected, or only partially performed, the febrile symptoms have subsided with greater reluctance, and the *specific* powers of the acid have at first appeared equivocal. Its action on the bowels is dissimilar to that of other acids; it often represses diarrhœa by correcting the putrescent diathesis, which should be considered as the principal cause of *diarrhœa* in fevers.

To illustrate the truth of these effects, I have subjoined a few cases:—

### Case I. *Scarlatina Anginosa.*

Elizabeth Garret, æt. 29, was admitted an in-patient of the Westminster-Hospital, on the 13th of July: it was the 4th day of fever, and the scarlet eruption was extremely vivid. She complained of great prostration of strength, thirst, head-ache; her deglutition was painful and distressing; her tongue was greatly furred; the animal temperature preternaturally exalted; and the pulse 136, with a weak beat. I immediately ordered her a purging-draft, composed of an *infusion of senna*, with *tartrate of potash*; after which I directed the exhibition of *muriatic acid*, in the proportion and manner above described. On the morning of the 14th, I found the pain of her head much relieved, the tongue less loaded, the thirst assuaged, the deglutition easier, and the pulse reduced to 128. In the evening of this day, all the febrile symptoms had still farther subsided, and the pulse was only 112. On the 16th, her throat was perfectly well; and, on the 18th, I found her her destitute of every febrile symptom, her pulse being 84. I then ordered a decoction of the bark, which, with the assistance of change of air, soon re-established her health.

CASE II. *Scarlatina Anginosa.*

Maria Holloway, æt. 9, was admitted into the Westminster-Hospital, on the 16th of July: she was suffering with every symptom of "*scarlatina anginosa.*" Her pulse was intermittent, and too frequent to be counted. I immediately directed the exhibition of muriatic acid, having first prescribed a saline evacuant. Her throat, in six hours, became much cleaner; the pulse was full, regular, and 130. In the course of four days, the febrile symptoms had subsided; her pulse having fallen, by regular and successive gradations, from 130 to 86 beats.

CASE III. *Typhus.*

Ann Thompson, æt. 36, had been laboring under the most aggravated symptoms of typhus fever, when I saw her in a wretched and filthy apartment, in the closest part of Westminster. The weather was extremely warm; and every collateral circumstance induced me to believe that the hand of Death could not be averted, even by the most judicious and powerful interposition of the medical art. Her pulse could be scarcely felt; a *low muttering* delirium, with cold extremities, all argued the dejected state to which the vital energy of the body was reduced. I directed the room to be well ventilated, and prescribed, as in the former cases, a saline purge; after which, a quart of diluted acid was prepared for her common beverage. In less than twenty-four hours, her pulse became more distinct; the delirium appeared less continued, and her feet grew warm. On the following day, I ordered a pint of port wine to be given, and insisted upon the steady use of the acid. In a few days her pulse was 120, much stronger and regular; her tongue was less black, and every febrile symptom had decreased. By a rigid adherence to the copious exhibition of the acid, the tongue became perfectly clean: I then administered the bark, and soon had the satisfaction of seeing my patient rapidly recovering from the debilitated state, in which so malignant a fever had necessarily left her.

Mr. Sutherland, to whom I acknowledge myself indebted for much practical information on this subject, has cured some hundred patients of "*typhus*" and "*scarlatina,*" in St. Margaret's workhouse, by means of a copious exhibition of muriatic acid, in which no wine was administered.

I remain, &c.

JOHN AYRTON PARIS.

*Westminster, Dec.* 7, 1809.

